# Epidemiology of cryptococcal meningitis and fluconazole heteroresistance in *Cryptococcus neoformans* isolates from a teaching hospital in southwestern China

**DOI:** 10.1128/spectrum.00725-24

**Published:** 2024-07-15

**Authors:** Yunfei Huang, Yongqi Zhang, Shuangshuang Yang, Hongling Lu, Hanbing Yu, Xingyue Wang, Xiaojiong Jia, Dijiao Tang, Linhong Wu, Shifeng Huang, Ping Yang

**Affiliations:** 1 Department of Clinical Laboratory Medicine, The First Affiliated Hospital of Chongqing Medical University, Chongqing, China; 2 Department of Laboratory Medicine, Fujian Key Clinical Specialty of Laboratory Medicine, Women and Children’s Hospital, School of Medicine, Xiamen University, Xiamen, China; 3 Division of Allergy and Clinical Immunology, Brigham and Women’s Hospital, Harvard Medical School, Boston, Massachusetts, USA; The Hebrew University-Hadassah School of Dental Medicine, Jerusalem, Israel

**Keywords:** cryptococcal meningitis, *Cryptococcus neoformans*, fluconazole, heteroresistance, antifungal susceptibility

## Abstract

Cryptococcal meningitis (CM), a common and serious opportunistic infection mostly caused by *Cryptococcus neoformans*, is primarily treated with fluconazole. Nevertheless, *Cryptococcus neoformans* strains that undergo repeated exposure to azoles can gradually acquire heteroresistance to fluconazole. The management of this specific CM infection poses a substantial challenge. Determining a globally accepted definition for fluconazole heteroresistance and developing effective and prompt methods for identifying heteroresistance is of utmost importance. We collected data on the clinical and epidemiological characteristics of patients diagnosed with CM. All the available *Cryptococcus neoformans* strains isolated from these patients were collected and subjected to antifungal susceptibility testing and evaluation of fluconazole heteroresistance. AIDS was present in 40.5% of the patients, whereas 24.1% did not have any underlying diseases. Patients with chronic diseases or impaired immune systems are susceptible to infection by *Cryptococcus neoformans*, a fungus that frequently (39.6%, 19/48) shows heteroresistance to fluconazole, as confirmed by population analysis profile (PAP).

**IMPORTANCE:**

Fluconazole heteroresistance poses a significant threat to the efficacy of fluconazole in treating cryptococcal meningitis (CM). Unfortunately, the standard broth microdilution method often misses the subtle percentages of subpopulations exhibiting heteroresistance. While the population analysis profile (PAP) method is esteemed as the gold standard, its time-consuming and labor-intensive nature makes it impractical for routine clinical use. In contrast, the Kirby-Bauer (KB) disk diffusion method offers a simple and effective screening solution. Our study highlights the value of KB over PAP and minimum inhibitory concentration (MIC) by demonstrating that when adjusting the inoculum concentration to 1.0 McFarland and subjecting samples to a 72-hour incubation period at 35°C, the KB method closely mirrors the outcomes of the PAP approach in detecting fluconazole heteroresistance. This optimization of the KB method not only enhances assay efficiency but also provides a blueprint for developing a timely and effective strategy for identifying heteroresistance.

## INTRODUCTION

Cryptococcal meningitis (CM) is a prevalent and serious opportunistic infection caused by *Cryptococcus neoformans* or, less commonly, *Cryptococcus gattii*. *Cryptococcus neoformans* primarily infect patients with HIV/AIDS (1, 2). The global annual estimated death toll from CM is 181,100, with the majority occurring in sub-Saharan Africa (2). The gold standard for treating CM involves induction therapy with amphotericin B combined with flucytosine, followed by consolidation and maintenance therapy with fluconazole (3). The mortality rates among patients with CM remain high despite the administration of antifungal therapy, with a 2-week mortality rate of around 20% and a 10-week mortality rate of approximately 35% (4). What’s worse, amphotericin B and flucytosine may not be affordable in many low-resource countries, especially in sub-Saharan Africa, where fluconazole is the only accessible antifungal drug (5). Consequently, fluconazole monotherapy is used in the majority of the countries. However, fluconazole monotherapy remains less effective even when relatively high doses of 800 to 1,200 mg/day are used. In a study cohort conducted in Africa, the mortality rates linked to the administration of fluconazole surpassed 50%–60% at the 10-week mark and reached 70% after 1 year (6, 7).

Primary fluconazole resistance has not been considered a major clinical problem because *Cryptococcus neoformans* generally exhibit low minimum inhibitory concentration (MIC) to fluconazole in extensive epidemiological surveys (8). However, the recurrence of *Cryptococcus neoformans* with a high MIC to fluconazole has been reported to result in clinical failure (9). Recent data suggest that fluconazole resistance (i.e., MIC > 8 mg/L) is as high as 10% in the primary isolates and 24% in the relapse isolates (10).

The potential failure of clinical treatment in cryptococcosis is attributed to the emergence of azole-resistant or heteroresistant mutants, as these strains possess the ability to overcome the therapeutic efficacy of azole antifungals (11–15). Heteroresistance refers to the variation in the level of antibiotic resistance among different subpopulations within a microbial population. This means that some clones may be resistant while others are susceptible (16). There is an adaptive stress mechanism that can enhance the survival ability of microorganisms under the pressure of antifungal drugs (14). An isolate may be classified as heteroresistant when the lowest antibiotic concentration giving maximum growth inhibition is eightfold higher than the highest non-inhibitory concentration, as described by El-Halfawy et al. (17). Heteroresistance is usually not detected using the standard broth microdilution method. Failure to detect heteroresistance may lead to the misclassification of non-susceptible strains as susceptible in clinical microbiology laboratories (17). This impacts the results of fluconazole sensitivity, potentially leading to inaccurate clinical decisions when utilizing fluconazole to treat CM.

In this study, we examined the epidemiological characteristics of 79 patients diagnosed with CM in our hospital from 2014 through 2023. We also analyzed heteroresistance to fluconazole in all the available clinical *Cryptococcus neoformans* strains isolated from these patients. This study was done to improve our understanding of the clinical features and prognosis of CM and fluconazole heteroresistance in *Cryptococcus neoformans*. We want to promote a uniform definition of fluconazole heteroresistance and support the development of efficient and rapid methods for detecting fluconazole heteroresistance.

## MATERIALS AND METHODS

### Cryptococcal strains and clinical data

Clinical data, including gender, age, and underlying diseases, were collected from patients diagnosed with CM at the First Affiliated Hospital of Chongqing Medical University from 2014 to 2023. Additionally, a total of 48 clinical isolates of *Cryptococcus* were collected. The identification of all isolates was conducted using the VITEK-2 Compact (bioMérieux, Marcy-l'Étoile, France) and VITEK MS (bioMérieux, Marcy-l'Étoile, France) systems. Control strains utilized in the study included *Candida albicans* ATCC 14053 and *Candida glabrata* ATCC MYA-2950. All strains were ultimately verified to be *Cryptococcus neoformans*. Before initiating the experiment, the strains were purified by isolating individual colonies on yeast extract peptone dextrose (YPD) agar plates (HKM, Guangdong, China) to guarantee their purity and viability. All clinical isolates were stored in a freezing medium containing 20% glycerol at −80°C in a refrigerator (Solarbio, Beijing, China).

### Antifungal drugs

Fluconazole (Meilunbio, Dalian, China) was dissolved in dimethyl sulfoxide (Solarbio, Beijing, China) to generate a stock solution with a concentration of 51,200 mg/L. The solution was subsequently filtered through a 0.22 µm filtration membrane (Biosharp, Hefei, China), dispensed, and then stored in sealed packages at −80°C.

Fluconazole disks (25 µg/disk) were obtained from Liofilchem (Roseto degli Abruzzi, Italy).

### Susceptibility testing

The broth microdilution method was conducted in accordance with the guidelines outlined in the Clinical and Laboratory Standards Institute (CLSI) M27-2017 (18) and M60-2020 (19). The fluconazole stock solution was diluted by the RPMI 1640 medium (Gibco, Carlsbad, CA, USA), which was pH-buffered to 7.0 with MOPS (Solarbio, Beijing, China), and was subsequently filtered through 0.22 µm membrane filters (Biosharp, Hefei, China). Fluconazole was subjected to a twofold dilution process, and 0.1 mL of fluconazole at various concentrations was sequentially dispensed into 96-well microdilution plates with U-shaped wells (Labselect, Hefei, China), resulting in a final fluconazole concentration range of 0.125–128 mg/L. An inoculum of *Cryptococcus neoformans* was prepared by suspending 3 to 5 colonies in sterile saline. The inoculum was standardized to a turbidity of 0.5 McFarland using a spectrophotometer (DensiCHEK Plus, bioMérieux, Marcy-l'Étoile, France). The inoculum was then diluted with RPMI 1640 medium. A volume of 0.1 mL of yeast suspension was added to each well of the 96-well microdilution plates with U-shaped wells, resulting in a final inoculum ranging from 0.5 × 10^3^ to 2.5 × 10^3^ CFU/mL. Subsequently, the plate was placed in an incubator at 35°C. The MIC of fluconazole was determined as the lowest concentration at which a 50% reduction in growth (significant decrease in turbidity) was observed compared to the growth control without the drug. The MIC was assessed visually after 24 and 48 hours of incubation for the quality control strain *Candida parapsilosis* ATCC 22019 and after 72 hours of incubation for *Cryptococcus neoformans*. Three replicates were performed for each concentration of fluconazole, in addition to the positive and negative controls. The fluconazole MIC of the quality control strains conformed to the criteria specified in the CLSI guidelines. The antifungal susceptibility testing was repeated twice, and any abnormal results were confirmed through three repetitions.

The susceptibility of the *Cryptococcus neoformans* isolates to fluconazole was assessed through the Kirby-Bauer (KB) disk diffusion method, which used Mueller-Hinton agar (Solarbio, Beijing, China) supplemented with 2% anhydrous glucose (Macklin, Shanghai, China) and 0.5 mg/L methylene blue (Solarbio, Beijing, China). The procedure followed the guidelines outlined in CLSI M44-2018 (20) and M60-2020 (19). The pH of the agar fell within the range of 7.2 to 7.4 at room temperature. The same procedure was employed to prepare the inoculum for both the KB disk diffusion method and the broth microdilution method, resulting in a final yeast suspension stock of 1 × 10^6^ to 5 × 10^6^ CFU/mL. A sterile cotton swab was immersed in the adjusted yeast suspension, firmly pressed against the inner wall of the tube to remove excess liquid, and then used to streak the entire agar surface thrice. The plate was rotated approximately 60° each time to ensure the uniform distribution of the inoculum across the plate. After allowing the plate to dry for a minimum of 3 minutes but no more than 15 minutes, the fluconazole disk was placed on the inoculated agar and gently pressed to ensure full contact with the surface. Measurements were taken after 48 hours of incubation at 35°C to determine the diameters of the inhibition zones. The results were interpreted in accordance with the guidelines of the CLSI.

### Screening of fluconazole-heteroresistant strains

The KB disk diffusion method was utilized to detect the heteroresistance phenotype of *Cryptococcus neoformans* to fluconazole. While incubating the KB disk to evaluate drug sensitivity in *Cryptococcus neoformans*. It is important to carefully notice any growth of colonies within the zones of inhibition. Strains with significant colony growth within the zone of inhibition should be interpreted as potentially heteroresistant strains (17). Furthermore, the study investigated the impact of inoculum concentration, incubation time, and incubation temperature on the evaluation of the KB disk diffusion method for detecting heteroresistance.

### Effect of the yeast suspension concentration on the presentation of heteroresistance phenotypes

The study investigated the effect of yeast suspension concentration on the presentation of heteroresistance phenotypes at three distinct concentration levels: 0.5, 1.0, and 2.0 McFarland standard turbidity.

### Effect of incubation temperature on the appearance of heteroresistance phenotypes

The study investigated the effect of incubation temperature on the appearance of heteroresistance phenotypes at three distinct temperatures: 28°C, 30°C, and 35°C.

### Effect of incubation time duration on the demonstration of heteroresistance phenotypes

The study investigated the effect of incubation time duration on the demonstration of heteroresistance phenotypes at two distinct times: 48 hours and 72 hours.

### Population analysis profile

The population analysis profile (PAP) method is a relatively quantitative approach used for colony counting and is widely recognized as the gold standard for assessing heteroresistant strains (17). The method described by El-Halfawy et al. (17) was used for the analysis of heteroresistance to fluconazole. The yeast suspension with a 0.5 McFarland standard turbidity was prepared using the method described above. It was then subjected to a 10-fold gradient dilution to achieve a yeast suspension with a concentration ranging from approximately 1 × 10^3^ to 1 × 10^6^ CFU/mL. A 50 µL aliquot of the yeast suspension was evenly distributed on YPD plates, either without fluconazole or with fluconazole at concentrations ranging from 4 to 256 mg/L across seven concentration gradients. This ensured that the number of colonies on each YPD plate fell within the range of 100 to 300 for easy counting. Three sets of replicates were performed for each concentration of YPD plates, followed by incubation at 28°C for 3 to 7 days. The number of colonies was graphed as log_10_ CFU/mL against the fluconazole concentration using the GraphPad Prism 9.5 software (GraphPad Software, La Jolla, CA, USA). All strains of *Cryptococcus neoformans* underwent PAP experiments to accurately assess the heteroresistance of *Cryptococcus neoformans* to fluconazole in this study.

### Stability of fluconazole heteroresistance *in vitro*

The subpopulation that showed resistance on the YPD agar plate with the highest concentration of fluconazole in the PAP assay was isolated for sequential passages on fluconazole-free YPD plates. Heteroresistant strains C1, C5, C7, C34, C37, and C42 were selected for fluconazole MIC determination using the broth microdilution method at five-generation intervals. Additionally, the KB disk diffusion method was used to measure the diameters of the inhibitory zones and to observe the growth of colonies within these zones. The study aimed to compare the growth of yeast colonies within the zones of inhibition before and after passages to evaluate changes in drug resistance and investigate the stability of heteroresistance.

## RESULTS

### Demographic characterization of CM

A total of 48 strains from 39 of the 79 patients were included in this study. There were 56 male and 23 female patients, with ages ranging from 23 to 83 years and a median age of 51 years. Of the 79 patients, 32 had AIDS, 19 had no underlying disease, and seven died between admission and discharge. Eighteen patients had hypertension, 13 had liver diseases, nine had diabetes mellitus, five were infected with tuberculosis, seven had long-term use of immunosuppressants and/or hormones, five had malignant tumors, three had kidney diseases, and two had autoimmune diseases. Among the 79 cases of CM, 72 patients experienced headache, 40 had fever, 48 reported nausea and vomiting, 26 showed signs of impaired consciousness, 34 exhibited positive signs of meningeal irritation, four had concurrent cryptococcal pneumonia, and seven had cryptococcemia (Table 1). Out of the 48 strains, four were serial strains (i.e., strains isolated simultaneously from various samples from distinct body sites in the same patient). A total of 44 strains were isolated from cerebrospinal fluid specimens, and four strains were isolated from blood.

**TABLE 1:** Demographics and clinical profile of patients with cryptococcus meningitis

Variable	n (% of available data)
Age (years), median, and IQR^a^	51 (49–64)
Gender	
Male	56 (70.9)
Female	23 (29.1)
AIDS	32 (40.5)
Underlying disease	
Hypertension	18 (22.8)
Liver diseases	13 (16.5)
Diabetes mellitus	9 (11.4)
Tuberculosis	5 (6.3)
Long-term immunosuppressants and/or hormones	7 (8.9)
Malignant tumors	5 (6.3)
Kidney diseases	3 (3.8)
Autoimmune diseases	2 (2.5)
Organ transplants	0
No underlying disease	19 (24.1)
Death	7 (8.9)
Clinical manifestations	
Headache	72 (91.1)
Fever	40 (50.6)
Nausea and vomiting	48 (60.8)
Disturbance of consciousness	26 (32.9)
Meningeal irritation signs (+)	34 (43.0)
Comorbidities	
Cryptococcal pneumonia	4 (5.1)
Cryptococcemia	7 (8.9)

^a^ IQR = interquartile range.

### Susceptibility analysis

**Fig 1:**
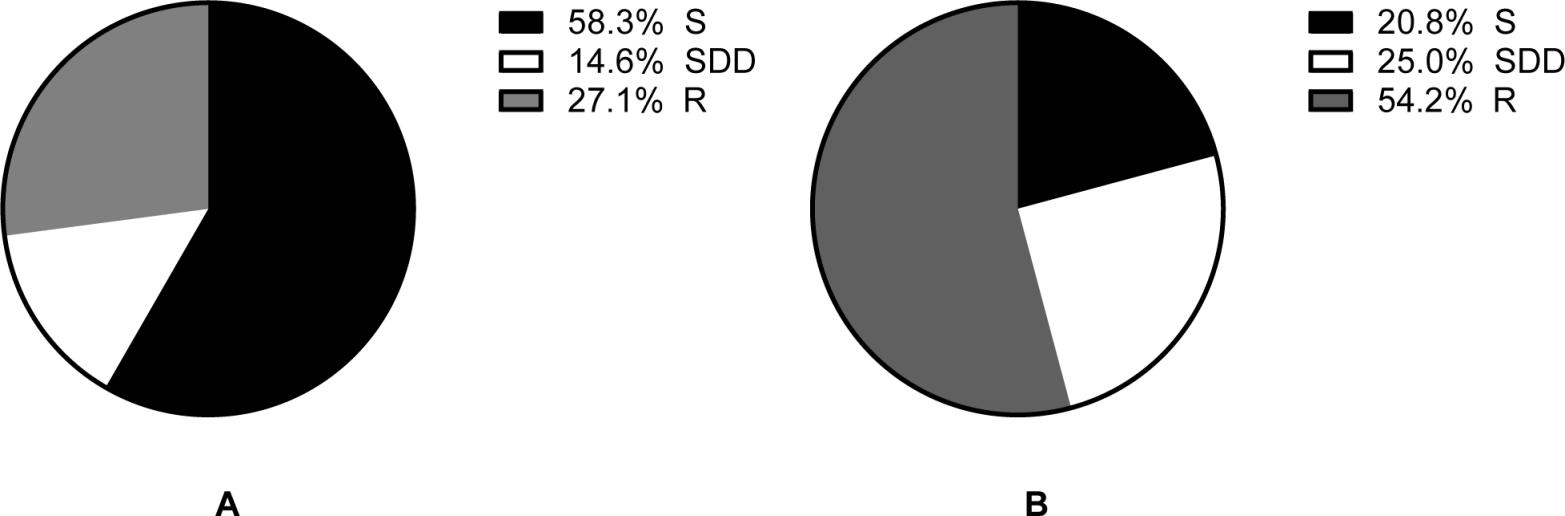
Influence of the incubation time durations on fluconazole susceptibility testing. The susceptibility of the 48 strains to fluconazole was assessed by measuring the diameters of the inhibition zones on the fluconazole disk using the KB disk diffusion method. The yeast suspension concentration was 0.5 McFarland, and the results were interpreted following the guidelines of CLSI M44-2018 (fluconazole S, ≥20 mm; SDD, 15–19 mm; R ≤ 14 mm) (20). (**A**) Fluconazole susceptibility testing results determined at 48 hours of incubation. (**B**) Fluconazole susceptibility testing results determined at 72 hours of incubation.

The determination of fluconazole susceptibility relied on the epidemiological cutoff values (ECV) as there has been no established clinical threshold for this purpose (24). The ECV for *Cryptococcus neoformans* var. grubii is defined as 8 mg/L and was used here. All 48 strains of *Cryptococcus neoformans* with fluconazole MIC ranging from 1 to 8 mg/L in this study were found to be wild type (WT). After extending the incubation time to 72 hours and measuring the diameters of the inhibition zones, it was observed that 48 strains of *Cryptococcus neoformans* showed a decrease in diameter compared to the measurement at 48 hours. This led to a decrease in the number of drug-sensitive yeast and an increase in the number of intermediate and drug-resistant yeast. When the incubation period was set to 48 hours, KB disk diffusion result indicated that out of the 48 strains, 28 (58.3%) were found to be sensitive (S), 7 (14.6%) were susceptible dose-dependent (SDD), and 13 (27.1%) were resistant (R) (Fig. 1A). When the incubation time was extended to 72 hours, there were 10 S strains, 12 SDD strains, and 26 R strains, constituting 20.8%, 25.0%, and 54.2% of the total, respectively (Fig. 1B).

### Screening for heteroresistant strains at CLSI recommended conditions

When the inoculum suspension concentration was 0.5 McFarland and the plates were incubated for 48 hours following CLSI recommendations, four potential heteroresistant strains were screened out using the KB disk diffusion method. These four strains were then evaluated using the PAP method, and three of them (3/4, 75.0%) were identified as fluconazole-heteroresistant strains.

### Impact of inoculum suspension concentration and incubation time duration on the presentation of heteroresistance phenotype

**Fig 2:**
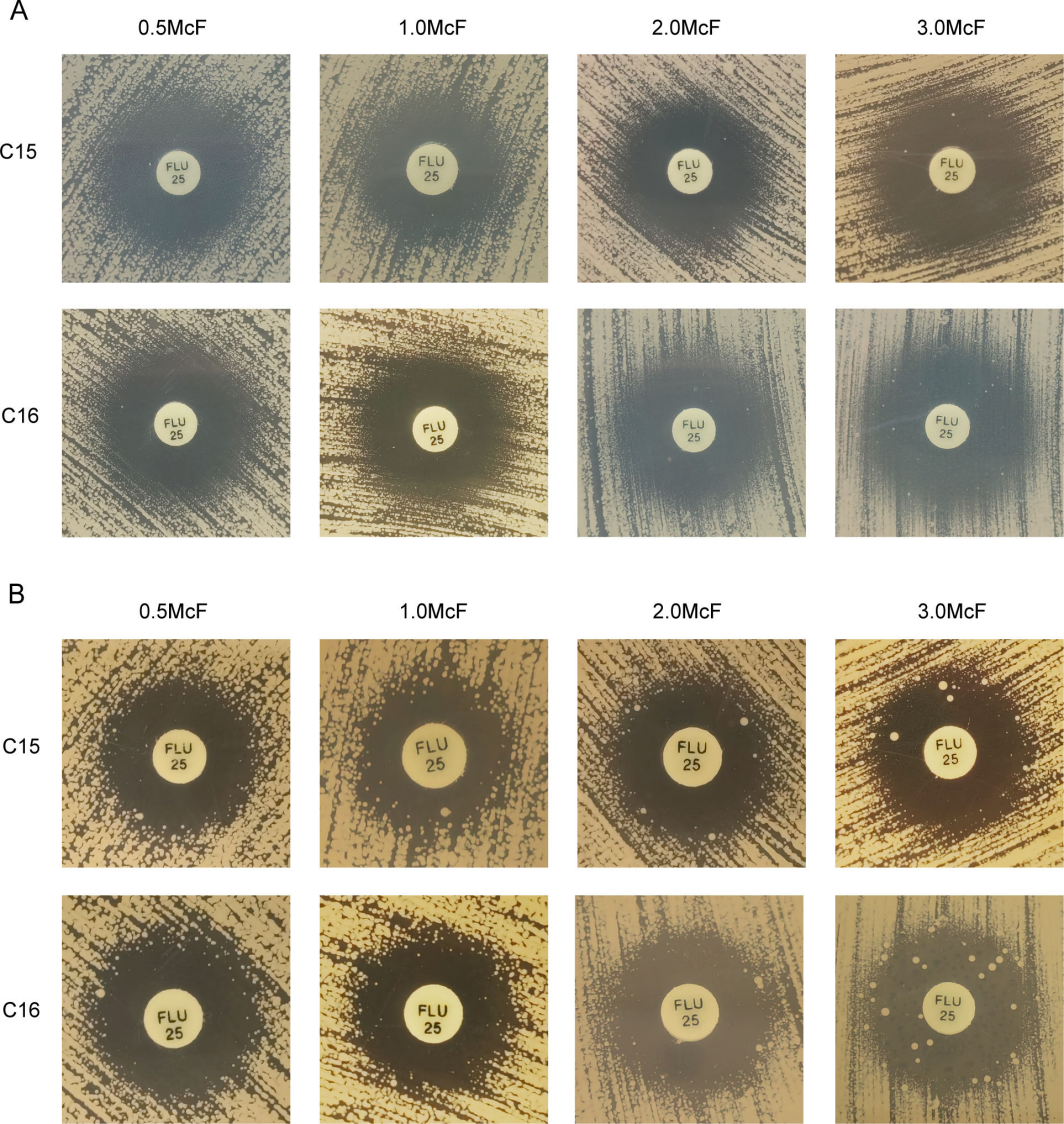
Impact of varying variances on the detection of fluconazole heteroresistance using the KB disk diffusion method. (**A**) When the incubation temperature was 35°C and the incubation time duration was set as 48 hours, the number of subclones in the zone of inhibition rose with the higher concentration of the inoculated yeast suspension. (**B**) Extending the incubation time to 72 hours resulted in a more pronounced heteroresistance phenotype as the concentration of the inoculated yeast suspension grew.

We conducted additional research to investigate how the concentration of inoculum suspension, incubation time duration, and incubation temperature affect the detection of heteroresistance. After extending the incubation period of the plates to 72 hours, 14 strains exhibited potential heteroresistance. Among them, 8 out of 14 (57.1%) were confirmed to be heteroresistant by the PAP method. Using the same method, 66.7% (8/12) of the heteroresistant strains were confirmed with an inoculum suspension adjusted to 1.0 McFarland and at an incubation duration of 48 hours. Furthermore, extending the incubation period to 72 hours resulted in the confirmation of 60.0% (12/20) of heteroresistant strains. At an inoculum suspension of 2.0 McFarland, 54.6% (6/11) of the strains exhibited heteroresistance after 48 hours of incubation, and 52.4% (11/21) showed heteroresistance after 72 hours (Table 2). Notably, the heteroresistance phenotype was observed after 48 hours of incubation, and the number of subclones within the zone of inhibition increased with higher yeast inoculum concentrations (Fig. 2A). Extending the incubation time to 72 hours revealed a more pronounced development of subclones within the zone of inhibition, indicating the heteroresistance phenotype (Fig. 2B). Furthermore, certain subclones that were not seen within the zone of inhibition at 48 hours of incubation were observed after 72 hours.

**TABLE 2:** Screening and confirmation of heteroresistant strains with varying variances^*d*^

PAP^*b*^ KB^*a*^ KB^*a*^	48 hours			72 hours		
	0.5McF	1.0McF	2.0McF	0.5McF	1.0McF	2.0McF
P1-C1^HR^						
P2-C2						
P3-C3						HR’
P4-C4^HR^			HR’			HR’
P5-C5^HR^					HR’	
P6-C6						HR’
P7-C7^HR^						
P8-C8		HR’	HR’			HR’
P8-C9^HR^				HR’	HR’	HR’
P9-C10^HR^	HR’	HR’	HR’	HR’	HR’	HR’
P10-C11						
P11-C12						
P12-C13				HR’	HR’	HR’
P13-C14^HR^						
P14-C15^HR^		HR’	HR’	HR’	HR’	HR’
P14-C16^HR^	HR’	HR’	HR’	HR’		HR’
P15-C17^HR^		HR’			HR’	HR’
P16-C18					HR’	
P17-C19						
P18-C20						HR’
P19-C21						
P20-C22				HR’		
P21-C23^HR^		HR’	HR’	HR’	HR’	HR’
P22-C24						
P23-C25						
P24-C26	HR’	HR’	HR’	HR’	HR’	HR’
P24-C27		HR’	HR’	HR’	HR’	
P25-C28		HR’	HR’		HR’	HR’
P26-C29						
P27-C30						
P27-C31					HR’	
P28-C32^HR^					HR’	HR’
P28-C33^HR^					HR’	
P29-C34^HR^	HR’	HR’				
P30-C35					HR’	
P31-C36^HR^		HR’	HR’	HR’	HR’	HR’
P32-C37^HR^						
P33-C38^HR^		HR’		HR’	HR’	HR’
P33-C39^HR^					HR’	
P34-C40						HR’
P34-C41						
P35-C42^HR^				HR’	HR’	HR’
P36-C43			HR’			HR’
P37-C44						
P37-C45				HR’	HR’	HR’
P38-C46						
P38-C47						
P39-C48				HR’		
**KB^HR’^**	4	12	11	14	20	21
**PAP^HR^**	3	8	6	8	12	11
**％HR^*c*^**	75.0%	66.7%	54.6%	57.1%	60.0%	52.4%

^*a*^ Heteroresistance was assessed using the KB disk diffusion method at an incubation temperature of 35°C.

^*b*^ Determination of heteroresistance in strains using the PAP method.

^*c*^ The ratio of PAP-confirmed heteroresistance strains to all phenotypically heteroresistant isolates screened out by the KB disk diffusion method.

^*d*^ HR = Heteroresistance strains as determined by the PAP method. HR’ = Strains determined to exhibit heteroresistance by the KB disk diffusion method. "P" = patients, with the number representing the specific patient from whom the strain originated. In total, we collected 48 strains from 39 patients.

### Impact of incubation temperature on the manifestation of heteroresistance phenotype

**Fig 3:**
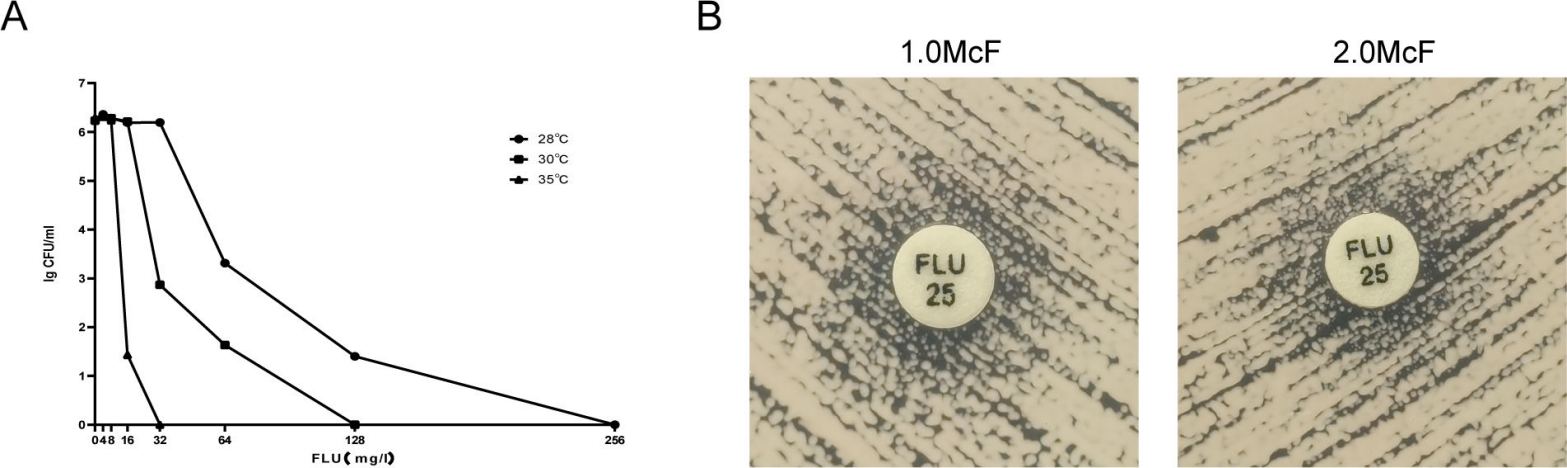
Effect of incubation temperature on heteroresistance expression. (**A**) Strain C37 was chosen for the experiment. The PAP method was used to evenly coat the yeast suspension on YPD agar medium. The medium was prepared fluconazole-free or with fluconazole at concentrations of 4–256 mg/L in seven dilutions. Colonies were enumerated following incubation at three distinct temperatures (28°C, 30°C, and 35°C) over a period ranging from 3 to 7 days. The GraphPad Prism 9.5 software was used to graph the number of colonies against the fluconazole concentration. Each data point on the graph represents the average value obtained from three separate experiments. (**B**) *Cryptococcus neoformans* displayed a resistant phenotype at 28°C across various inoculum concentrations.

One specific strain exhibited no colony growth when exposed to 32 mg/L fluconazole at 35°C on YPD plates. Similarly, no colony development was seen on YPD plates containing 128 mg/L fluconazole at 30°C and on plates with 256 mg/L fluconazole at 28°C (Fig. 3A). Nevertheless, after examining the heteroresistance phenotype using the KB disk diffusion method at a temperature of 28°C, it was observed that the strain displayed resistance at varying degrees of inoculum concentrations. The growth of colonies around the fluconazole disk is clearly shown in Fig. 3B.

### Prevalence of heteroresistance and phenotypic analysis of clinical isolates

**Fig 4:**
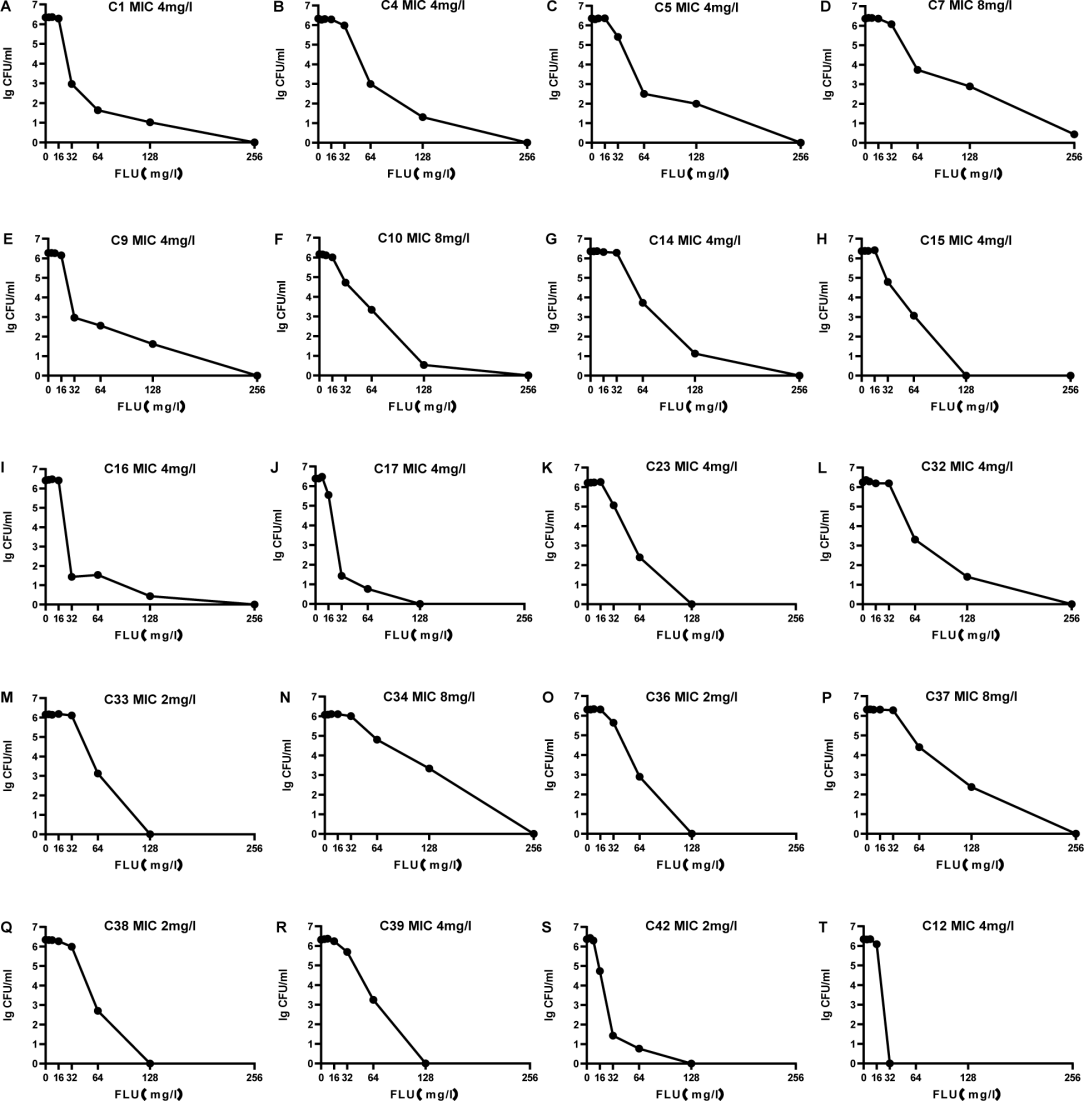
Results of the PAP experiment. (**A–S**) PAP curves were generated for 19 clinical isolates of *Cryptococcus neoformans* showing fluconazole heteroresistance. These strains exhibited the ability to grow on agar plates with different fluconazole concentrations, despite having identical MIC. (**T**) Strain C12 was determined to be non-heteroresistant based on the results of the PAP method. This was indicated by the curve dropping to zero shortly after surpassing the strain’s MIC.

A total of 48 strains were examined, with 39.6% (19/48) showing fluconazole heteroresistance. As fluconazole concentration grew, the number of resistant subclones in the parental strains cultivated on the plates decreased gradually. None of the heteroresistance subpopulations exhibited an MIC less than eight times lower than the parental strains (Fig. 4). Although all the 19 heteroresistant strains were determined as susceptible to fluconazole by the broth microdilution method, the PAP method results indicated varying susceptibility levels to fluconazole across the resistant subpopulations, with a few subpopulations capable of growing on fluconazole plates at 16 to 32 times the MIC. When evaluating the MICs of the subcolonies grown on plates containing the highest fluconazole concentration, it was noted that the maximum fluconazole concentration did not match the fluconazole MIC of the colonies. Nevertheless, all of them showed higher MIC values than the colonies grown on fluconazole-free plates, and the MIC of their resistant subpopulations increased by 8- to 16-fold compared to the parental strains. Heteroresistant subclones were found in 19 strains in this study, with frequencies ranging from 2.56E−06 to 1.84E−03 (Table 3).

**TABLE 3:** Characterization of heteroresistant strains of *Cryptococcus neoformans*

Strain	AIDS	Gender^*a*^	MIC^*b*^	FLU (PAP)^*c*^	MIC (PAP)^*d*^	Classification^*e*^	HR frequency^*f*^
C1	Yes	M	4	128	32	HR	1.20E−05
C4	No	F	4	128	64	HR	9.26E−06
C5	No	M	4	128	32	HR	4.42E−05
C7	Yes	F	8	256	64	HR	2.89E−06
C9	No	M	4	128	32	HR	2.48E−05
C10	No	M	8	128	64	HR	9.01E−06
C14	No	F	4	128	32	HR	1.47E−05
C15	No	M	4	64	32	HR	5.13E−04
C16	No	M	4	128	32	HR	2.56E−06
C17	Yes	M	4	64	32	HR	2.75E−05
C23	Yes	M	4	64	32	HR	1.63E−04
C32	No	M	4	128	64	HR	1.52E−05
C33	No	M	2	64	16	HR	1.12E−03
C34	No	F	8	128	64	HR	1.84E−03
C36	No	M	2	64	32	HR	3.91E−04
C37	No	M	8	128	128	HR	1.15E−04
C38	No	M	2	64	32	HR	2.42E−04
C39	No	M	4	64	32	HR	8.63E−04
C42	No	M	2	64	32	HR	2.79E−05

^*a*^ The patient’s gender is indicated by "M" for male and "F" for female.

^*b*^ The MIC (mg/L) of fluconazole for the parental strains was determined by the broth microdilution method.

^*c*^ The highest concentration of fluconazole (mg/L) at which heteroresistant subclones are able to proliferate on YPD plates in the PAP testing.

^*d*^ The MIC (mg/L) of the subcolonies that can grow on the YPD plate with the highest fluconazole concentration.

^*e*^ HR = heteroresistance.

^*f*^ The frequency of heteroresistant subclones in each of the 19 strains, which was calculated by dividing the number of colonies capable of growing on the YPD plate with the highest fluconazole concentration by the count of colonies on the fluconazole-free plate.

### Stability of heteroresistance *in vitro*

**Fig 5:**
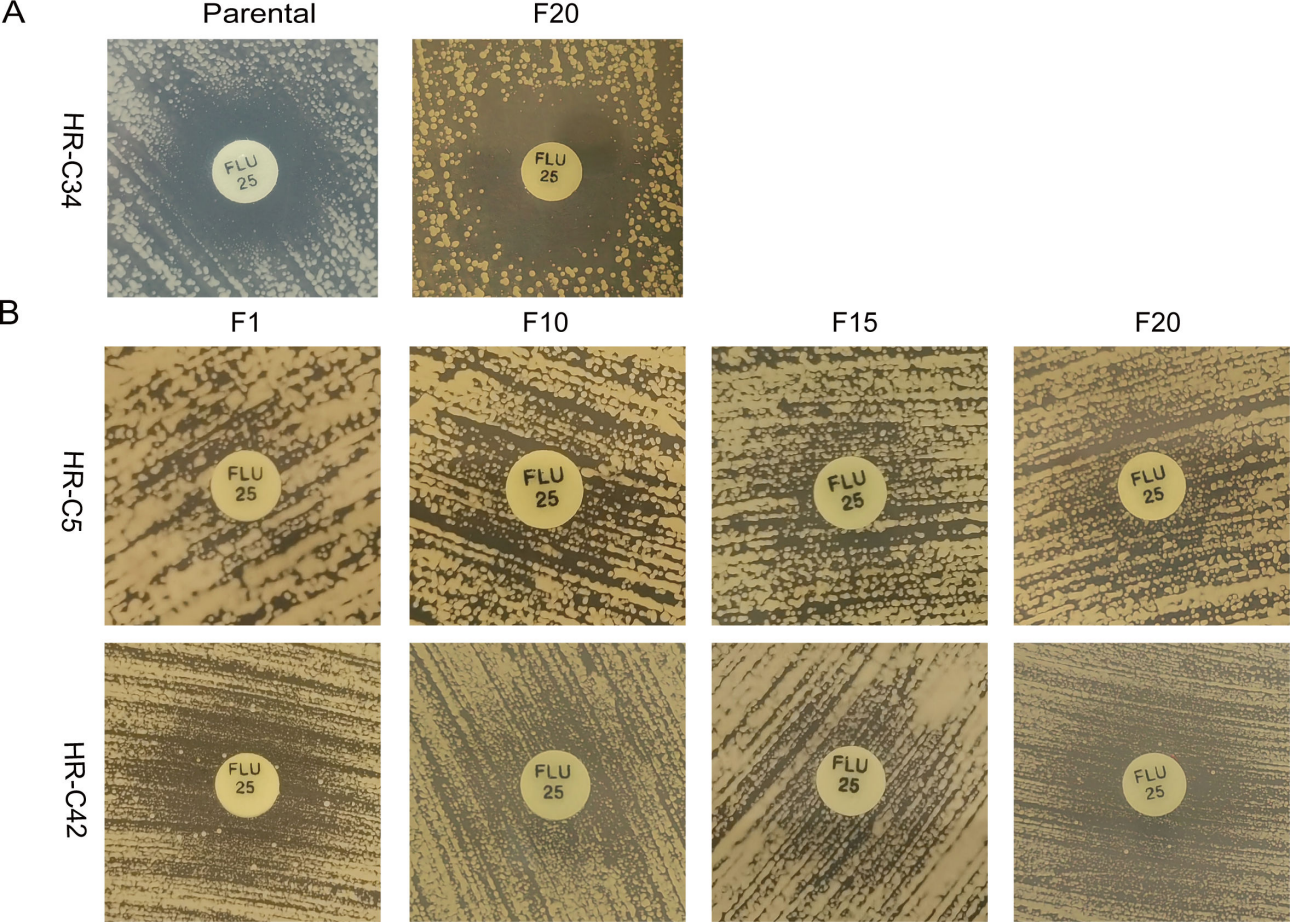
Experiments on the stability of heteroresistant strains. (**A**) After being passaged in the fluconazole-free medium for 20 generations, the diameter of the fluconazole zone of inhibition in the KB disk diffusion method assay of strain C34 returned to a size comparable to that of the parental strain. (**B**) Strains C5 and C42 consistently exhibited resistant phenotypes to fluconazole in the KB disk diffusion method assay for 20 generations, despite a steady drop in the MIC of the strains. Intriguingly, strain C42, when adjusted to a concentration of 2.0 McFarland in the yeast suspension, showed visible colonies surrounding the fluconazole disk, while still maintaining a resistant phenotype.

Six heteroresistant strains denoted as C1, C5, C7, C34, C37, and C42, were selected for *in vitro* stability analyses. Heteroresistant subpopulations of these six strains were isolated from YPD plates containing the highest fluconazole concentration using the PAP method. Subsequently, these subpopulations were serially passaged in a fluconazole-free YPD medium. The MICs of the heteroresistant subclones were evaluated every five generations using the broth microdilution method, while the phenotypic traits were examined using the KB disk diffusion method. The findings demonstrated that the heteroresistant subpopulation of all six tested strains exhibited instability during the passaging process. After 5 to 10 passages on a fluconazole-free medium, the strains showed a steady drop in fluconazole resistance, eventually reverting to the susceptibility level of the original strains by the 20th generation (Table 4). On the other hand, the diameters of the zones of inhibition for strains C34 and C37 returned to the original diameters of the zones of inhibition of the parental strains after 20 passages (Fig. 5A). Yet, the diameter of the inhibition zones for the remaining four strains maintained a drug-resistant phenotype even after 20 passages, contradicting the MICs of their respective parental strains. Interestingly, upon adjusting the yeast suspension concentration of strain C42 to 2.0 McFarland units, we conducted the KB disk diffusion method to assess the phenotypic traits of the ancestral subpopulation. Notably, colonies emerged around the fluconazole disk, indicating a resistant phenotype for strain C42. However, larger and more prominent colonies were observed surrounding the fluconazole disk (Fig. 5B).

**TABLE 4:** Stability of heteroresistant strains

Generation	MIC (mg/L) of fluconazole in heteroresistant subclones					
	C1	C5	C7	C34	C37	C42
F1	32	32	64	64	128	32
F5	32	16	64	32	64	16
F10	16	16	32	16	32	8
F15	8	8	8	8	8	4
F20	4	4	8	8	8	2

## DISCUSSION

In foreign countries, *Cryptococcus neoformans* infections are primarily associated with AIDS (21), whereas in our country, most reported *Cryptococcus neoformans* infections are associated with non-AIDS (22). The present study found that 79 patients with CM were predominantly male (70.9%). The median age was 51 years. Non-AIDS patients accounted for the majority (59.5%), while AIDS patients comprised the remaining minority (40.5%). However, most of the non-AIDS patients had underlying conditions such as diabetes mellitus, hypertension, liver disorders, tuberculosis, and malignant tumors. 24.1% of patients didn’t have AIDS or any underlying diseases, which is consistent with the findings of numerous studies both domestically and internationally (23–25). Our results indicated that individuals with chronic conditions or weakened immune systems are most susceptible to *Cryptococcus neoformans* infection. Nevertheless, individuals with normal immunological function are equally susceptible to *Cryptococcus neoformans* infection. A prior *in vitro* study, which analyzed approximately 3,000 strains of *Cryptococcus neoformans* from various geographic regions, reported a fluconazole resistance rate of 11.2% (26). This suggests a reduced susceptibility of *Cryptococcus* spp. to fluconazole. However, the MIC results from our study indicated that all *Cryptococcus neoformans* strains exhibited WT susceptibility (MIC < 8 mg/L) to fluconazole, implying the absence of associated resistance. Nevertheless, the issue of resistance remains a significant concern and requires ongoing attention.

In this study, we explored the impacts of inoculum concentration, incubation time, and temperature on detecting the heteroresistance phenotype using the KB disk diffusion method. Our findings revealed that for confirming heteroresistance in *Cryptococcus neoformans* via the PAP method, strains resistant to heteroresistance were better screened at an incubation temperature of 28°C.

In our investigation of heteroresistant strains using the KB disk diffusion method, we observed that detection of heteroresistance in *Cryptococcus neoformans* isolates to fluconazole was more consistent with the PAP method when inoculated with a yeast suspension at a concentration of 1.0 McFarland and then incubated for 72 hours at 35°C. However, we noted that most isolates exhibited subclones that continued to grow after 72 hours, and the diameters of the inhibition zones were smaller at 72 hours compared to 48 hours. This phenomenon might be attributed to fungal tolerance, a term commonly used to describe the ability of microorganisms to survive brief exposures to drug stress without altering the MIC (27).

As for the reasons for the higher hit rate by the KB method, the consistency observed between the KB and PAP methods when inoculated with a yeast suspension at 1.0 McFarland concentration and incubated for 72 hours at 35°C could be due to several factors. The KB method might have better captured the heteroresistant subpopulations due to its simplicity and effectiveness in screening, allowing for more accurate detection. Additionally, the extended incubation period at a higher temperature could have facilitated the growth of heteroresistant subpopulations, leading to a higher hit rate. However, further studies are warranted to fully assess the impact of fungal tolerance on the detection of heteroresistance in the KB disk diffusion method.

In general, the standard laboratory antifungal susceptibility tests, especially the standard broth microdilution method, are unable to detect the modest percentages of subpopulations that show heteroresistance. In the clinical microbiology laboratory, failure to recognize heteroresistance may result in misidentifying non-susceptible strains as susceptible (17). As a result, epidemiological investigations of MIC ignore heteroresistance, which leads to low rates of reported primary resistance. The presence of heteroresistance complicates the diagnosis and treatment of the infection. On the other hand, drug susceptibility results obtained from the KB disk diffusion method must be interpreted cautiously to prevent treatment failures caused by false positives and false negatives.

Previous studies have shown that in the KB disk diffusion method assay experiments, colonies that exhibit significant growth within a well-defined zone of inhibition indicate the presence of heteroresistance and could potentially be used as an alternative to the PAP method (17). The disparities observed between the two heteroresistance screening methods, namely the KB disk diffusion method and the PAP method, in our findings, could be attributed to variables such as methodological discrepancies, yeast concentration, and testing conditions (e.g., temperature). These factors contributed to the inconsistent detection of heteroresistant subpopulations of *Cryptococcus neoformans*. As a result, some false-positive and false-negative rates were noted in the KB disk diffusion method compared to the PAP method. However, the results from our study indicated that although the KB disk diffusion method is a direct and rapid screening method, it is relatively poorly characterized and cannot yet replace the PAP method for detecting heteroresistance in *Cryptococcus neoformans*. Furthermore, the lack of a standardized definition of heteroresistance could lead to the misclassification of heteroresistant strains as non-heteroresistant, which could hinder their clinical therapeutic assessment. Overall, there is a lack of standardized definitions for heteroresistance, as well as standard assays and global guidelines for determining heteroresistance (16, 17), leading to discrepancies in the results obtained from various methods and between different laboratories (28–30).

In our study, 39.6% (19/48) of *Cryptococcus neoformans* clinical isolates from patients with CM exhibited heteroresistance to fluconazole. The PAP method is regarded as the gold standard for detecting heteroresistance, but it is time-consuming, labor-intensive, and unlikely to be suitable for routine clinical examinations. Heteroresistance may go undetected by current clinical assays. The potential impact of heteroresistance includes treatment failure, relapse, and the formation of persistent chronic infections (17). Additionally, it contributes to the failure of fluconazole monotherapy for human CM (31). Therefore, there is a need to investigate the development of alternative simple and rapid methods for detecting heteroresistance.

The study’s limitation lies in its failure to assess the adaptation of heteroresistant-derived colonies or their stability under fluconazole subculturing, which mirrors potential clinical failures during drug therapy. Aneuploidy alterations, particularly duplication of chromosome 1, can lead to heteroresistance to fluconazole. However, we did not investigate chromosome 1 duplication in our study.

We observed a decrease in the MIC of the heteroresistant strains as the number of passages increased during our *in vitro* stability test. The MICs returned to the same level as their parental strain after approximately 20 generations. However, in the KB disk diffusion method, only two strains exhibited a zone of inhibition similar to the parental strain after 20 passages in fluconazole-free plates. In contrast, the zone of inhibition did not increase for the remaining four strains, and they consistently exhibited a resistant phenotype. Regrettably, we were unable to continue the process of passing down to the next generation owing to time constraints. It is unknown if the zone of inhibition in the KB disk diffusion method for antifungal susceptibility assay will be restored to the level of the parental strains as the number of passages increases, which is a limitation of this investigation. Our study was conducted *in vitro* and the heteroresistance was analyzed at 28°C. Additional investigation is needed to determine if variations in human body temperatures have an impact on heteroresistance. This study had a limited sample size and provided insufficient clinical information to fully comprehend the significance of heteroresistance in clinical outcomes, such as mortality. Heteroresistance, a phenomenon observed in microbial populations, denotes the presence of subpopulations with varying degrees of antibiotic resistance. This suggests the coexistence of both sensitive and resistant clones within the same culture (16). One school of thought posits that heteroresistance is intrinsic, existing prior to antibiotic exposure, wherein a resistant subpopulation coexists alongside sensitive ones, potentially proliferating by outcompeting fluconazole-sensitive counterparts (11, 14, 31). Conversely, an opposing view suggests that heteroresistance is induced by drug exposure. Proponents of this perspective argue that exposure to fluconazole triggers dynamic shifts in antifungal susceptibility, often linked with the development of aneuploidy (32). Both viewpoints carry clinical significance, with implications for the management and treatment outcomes of fungal infections. Indeed, the presence of heteroresistance may correlate with clinical treatment failure. Notably, *in vitro* detection of heteroresistance through culturing on fluconazole-containing agar could inadvertently exacerbate this phenomenon (31). Heteroresistance may also be misinterpreted when only one colony from the original population of colonies isolated from a patient is analyzed for susceptibility to antifungal agents (33). These factors may have hindered our study on heteroresistance to fluconazole in *Cryptococcus neoformans*.

In conclusion, fluconazole heteroresistance may significantly impact the effectiveness of fluconazole in treating CM. A more comprehensive understanding of the connection between fluconazole exposure and resistance is necessary to further optimize fluconazole-based treatment regimens for CM. Therefore, it is imperative to establish a standardized definition and a streamlined, simple, and rapid method for detecting heteroresistance. It is necessary to conduct additional research on the relationship between fluconazole heteroresistance and treatment failure in CM and to develop alternative methods for detecting heteroresistance.

## Data Availability

Data and materials used in this work are available from the corresponding author upon request.
